# An Approach to Integrate Metagenomics, Metatranscriptomics and Metaproteomics Data in Public Data Resources

**DOI:** 10.1002/pmic.202500002

**Published:** 2025-04-28

**Authors:** Shengbo Wang, Satwant Kaur, Benoit J. Kunath, Patrick May, Lorna Richardson, Alexander B. Rogers, Paul Wilmes, Robert D. Finn, Juan Antonio Vizcaíno

**Affiliations:** ^1^ European Molecular Biology Laboratory – European Bioinformatics Institute (EMBL‐EBI) Wellcome Genome Campus Hinxton Cambridge UK; ^2^ Systems Ecology Group Luxembourg Centre for Systems Biomedicine University of Luxembourg Esch‐sur‐Alzette Luxembourg; ^3^ Department of Life Sciences and Medicine Faculty of Science Technology and Medicine University of Luxembourg Esch‐sur‐Alzette Luxembourg; ^4^ Bioinformatics Core Luxembourg Centre for Systems Biomedicine University of Luxembourg Esch‐sur‐Alzette Luxembourg

**Keywords:** data integration, data workflow, metaproteomics, metagenomics, metatranscriptomics

## Abstract

The availability of public metaproteomics, metagenomics and metatranscriptomics data in public resources such as MGnify (for metagenomics/metatranscriptomics) and the PRIDE database (for metaproteomics), continues to increase. When these omics techniques are applied to the same samples, their integration offers new opportunities to understand the structure (metagenome) and functional expression (metatranscriptome and metaproteome) of the microbiome. Here, we describe a pilot study aimed at integrating public multi‐meta‐omics datasets from studies based on human gut and marine hatchery samples. Reference search databases (search DBs) were built using assembled metagenomic (and metatranscriptomic, where available) sequence data followed by de novo gene calling, using both data from the same sampling event and from independent samples. The resulting protein sets were evaluated for their utility in metaproteomics analysis. In agreement with previous studies, the highest number of peptide identifications was generally obtained when using search DBs created from the same samples. Data integration of the multi‐omics results was performed in MGnify. For that purpose, the MGnify website was extended to enable the visualisation of the resulting peptide/protein information from three reanalysed metaproteomics datasets. A workflow (https://github.com/PRIDE‐reanalysis/MetaPUF) has been developed allowing researchers to perform equivalent data integration, using paired multi‐omics datasets. This is the first time that a data integration approach for multi‐omics datasets has been implemented from public data available in the world‐leading MGnify and PRIDE resources.

**Abbreviations**
DB: database
ENA: European Nucleotide Archive
FAIR: findable, accessible, interoperable and reusable
FDR: false discovery rate
IGV: integrative genome viewer
INSDC: International Nucleotide Sequence Database Consortium
MAGs: metagenome assembled genomes
MS: mass spectrometry
PSMs: peptide spectrum matches
PUFs: proteins of unknown function
uHGG: unified human gastrointestinal genome

## 1 Introduction

The past two decades have witnessed the increasing application of culture‐independent omics methods such as metagenomics, metatranscriptomics and metaproteomics to facilitate the in‐depth study of microbial communities in a wide range of environments [1, 2, 3, 4, 5]. Although metagenomics provides information on the species diversity and functional potential of microbiomes, metatranscriptomics determines the genes actively transcribed at the point of sample collection. Meanwhile, metaproteomics resolves proteins from multiple organisms and has emerged as a powerful tool to identify and quantify the functions expressed within a given microbial community [6]. Although the analysis of metagenomic and metatranscriptomic datasets can be performed using existing reference databases (DBs), a typical (meta)proteomics analysis requires a tailored protein sequence DB to match the experimentally derived tandem mass spectra (MS/MS) against, in order to detect the proteins present in the sample [7], and to avoid random matches that might result from large DB searches like the NCBI non‐redundant DB. However, the quantity of peptide‐spectrum matches (PSMs) obtained (and thus the number of identified peptides and proteins) is largely dependent on the suitability of the search DB used. Indeed, an incomplete DB risks missing or falsely identifying proteins, while an excessively large DB decreases the sensitivity of the analysis, increases computation time and inflates the false discovery rate (FDR) [8].

High‐throughput DNA sequencing technologies have revolutionised the field of genomics, especially metagenomics, where their application has yielded unparalleled insights into microbial diversity. The application of metagenomic assembly has enabled the identification of millions of full‐length proteins, the majority of which are not represented in protein DBs such as UniProtKB [9]. Indeed, the MGnify proteins resource currently contains over 2.4 billion non‐redundant sequences, derived from a range of biomes. Although it may seem logical that such a DB could address the limited reference DB coverage when dealing with metaproteomics [10], as mentioned above, using (too) large reference DBs in metaproteomics analyses can lead to a lower sensitivity [11]. Thus, the ideal search DB for a metaproteomics analysis should represent the total coding potential of the organisms present in the sample. As such, the preferred approach in metaproteomics analyses is to generate a search DB using metagenomic and/or metatranscriptomic sequencing data generated from the very same samples [12, 13]. This strategy ensures that the DB closely reflects the specific microbial community of interest. Nonetheless, there are a multitude of metaproteomics studies when sample‐specific nucleotide sequence DBs are not generated. In such cases, search DBs are constructed based on the aggregation of publicly available gene/protein sequences originating from analogous samples.

Multi‐omics microbial data are available in a number of different bioinformatics resources. On one hand, MGnify (https://www.ebi.ac.uk/metagenomics) [14], is a freely available hub for the analysis, exploration and archiving of microbiome‐derived sequence data. The resource develops standardised analysis pipelines to provide taxonomic and functional profiles of user‐submitted or public data. MGnify also provides an assembly of metagenomic and metatranscriptomic data as a service, and generates metagenome‐assembled genomes (MAGs) for inclusion in biome‐specific genome catalogues.

On the other hand, PRIDE (https://www.ebi.ac.uk/pride/) is the largest repository of mass spectrometry (MS)‐based proteomics data worldwide [15], with the number of metaproteomics datasets available increasing significantly. One of the main aims of PRIDE is to reuse/reanalyse public proteomics datasets using reproducible open data pipelines, such that proteomics data may be integrated with other public ‘omics data types, and can be readily accessed by life scientists, including non‐experts in proteomics. This approach has already been successfully demonstrated involving bioinformatics resources such as Expression Atlas [16, 17, 18] (for protein abundance information) and UniProtKB (UniProt KnowledgeBase) [19, 20] (for post‐translational modification data). Data reanalyses serve to harmonise results across datasets and either confirm the results reported in the original publication, or provide new biological insights. Additionally, they enable access for the visualisation of proteomics data in other popular data resources.

Motivated by the increased popularity of ‘meta‐omics’ approaches and the growth in the availability of paired public datasets, this study aims to develop the workflows and methodologies necessary to integrate and visualise public metaproteomics data from PRIDE with associated metatranscriptomic and metagenomic data from MGnify. The further aim is to investigate the potential of generating (or supplementing) search DBs using the biome‐specific MGnify Genomes catalogues, as an alternative source of a reference DB. This pilot study examines three distinct multi‐omic datasets available in both PRIDE and MGnify, demonstrating how systematic integration of meta‐omic data from these two public resources can be achieved. Through this approach, we aim to illustrate the feasibility and benefits of integrating diverse meta‐omics datasets to enhance our understanding of microbial communities.

## 2 Methods

### 2.1 Selection of Datasets

Datasets in PRIDE were identified for re‐analysis within this study based on the following criteria: (i) human gut or environmental samples where the metaproteomic sample was processed as label‐free and non‐enriched for post‐translational modifications; (ii) the study contained paired metagenomic and/or metatranscriptomic data available via MGnify (https://www.ebi.ac.uk/metagenomics/) and/or the European Nucleotide Archive (ENA) [21]; (iii) data were generated using Thermo Fisher Scientific instruments; and (iv) metadata connecting the samples was available either in the original publication, or through contacting the authors. Of the resulting multi‐omics datasets, two human gut studies (short study titles: ‘healthy gut’ and ‘diabetes gut’) and one marine study (short study title: ‘marine hatchery’) were selected to demonstrate applicability across different biomes. These biomes were selected due to the availability of corresponding biome‐specific MGnify Genomes catalogues and associated protein catalogues. The ‘diabetes gut’ dataset was specifically selected due to the availability of both metagenomics and metatranscriptomics data. Accession numbers of the datasets in PRIDE, MGnify and ENA and main characteristics are summarised in Table 1.

**TABLE 1:** Description of the public multi‐omics datasets selected for the benchmarking study.

**Dataset Short name**	**Original PRIDE dataset**	**MGnify accession**	**ENA accession**	**Number of samples**	**Number of raw files**	**Short d description of the metaproteomics dataset**	**Types of sequencing data**	**# Assemblies**	**PMID publication**	**# contigs**	**# predicted CDS**
Healthy gut	PXD005780	MGYS00005657	ERP124921	15	15	Faecal samples from 15 healthy individuals	Metagenomic	37	28709472	647,668	1,388,306
Diabetes gut	PXD003791	MGYS00001985	ERP104047	36	108	Faecal samples from 18 individuals from four families with at least two members from each family having type I diabetes mellitus	Metagenomic and metatranscriptomic	220	27723761	4,564,628	10,963,109
Marine hatchery	PXD020692	MGYS00005863	ERP133749	36	36	Six water samples from hatchery at pH 7.1 and 8.2 and three time points	Metagenomic	6	33902744	2,673,652	5,523,591

The three selected metaproteomics datasets were manually curated to accurately map the MS raw files to the corresponding biological samples as this information was incomplete in PRIDE at the time of submission. Sample and experimental design information are provided using a SDRF (Sample Data Relationship File)‐Proteomics file [22]. The metaproteomics samples were then manually mapped to the corresponding metagenomics/metatranscriptomics samples in ENA/MGnify.

### 2.2 Generation of Protein Sequence Search DBs for Benchmarking

Metagenomic and metatranscriptomic raw‐sequence datasets were downloaded from the ENA, quality filtered using MGnify standard procedures and assembled using metaSPAdes v. 3.14.0 [23]. Contigs were filtered for length >500 bp from metagenomic assembly, and >200 bp from metatranscriptomic assembly. Protein coding sequences were identified in the assembled contigs using the standard MGnify combined gene caller that primarily utilises Prodigal, supplemented by non‐overlapping FragGeneScan predictions [14], to account for the likely existence of fragmented genes within the metagenomic datasets.

Protein sequences were aggregated to construct the search DBs according to the following strategies: (i) sequences from all assembled runs from a given study; (ii) sequences from all MAGs derived from a given study; (iii) sequences from all assemblies in a study and a set of genomes from the Unified Human Gastrointestinal Genome (UHGG) catalogue that were matched to the study (identified using sourmash); (iv) sequences from all MAGs derived from a given study and genomes from the UHGG catalogue matched to the study; (v) sequences from the pan‐genomes of all UHGG genomes from the same geographic continent as the study samples; and (vi) sequences from all UHGG genomes from the same geographic continent as the study samples. In the cases where both metagenomic and metatranscriptomic data were available from the same sample, we evaluated aggregating the predicted proteins from metagenomics and metatranscriptomics. We also compared search DBs containing proteins predicted by the combined gene caller versus those just predicted using Prodigal.

Briefly, the subsets of genomes from the UHGG that were used to generate DBs (strategies iii—vi mentioned above) were determined using Sourmash (v. 4.2.2) [24]. Sourmash sketches were also used in the scenario where the study contained many samples, making the specific search DBs (containing all samples included in the study) too large. For such cases, we used Sourmash to define groups of the most similar samples in the study, with predicted protein sequences from the sample groups aggregated to form search DBs. We also used a tree traversal algorithm to dynamically generate multiple search DBs. This method is described in detail under the ‘Data integration open workflow’ section.

All search DBs were supplemented with the human proteome (UniProt human reference proteome release‐2019_05, including isoforms, 95,915 sequences), the cRAP database of contaminants (https://www.thegpm.org/crap/, 115 protein sequences) and decoy sequences generated using reversed sequences of all protein entries in the DB.

### 2.3 Proteomics Raw Data Processing

All the three metaproteomics studies were reanalysed separately, using the software combinations described below, and utilising the search DBs generated from the metagenomics/metatranscriptomics data as described above. We first converted Thermo Raw files into the *mgf* format for mass spectra using ThermoRawFileParser [25], and conducted the metaproteomics searches to identify peptides and proteins using SearchGUI [26] (v. 3.3.20) with X!Tandem [27] and MS‐GF+ [28] as search engines. We used PeptideShaker [29] (v. 1.16.45) for the post‐processing steps, which combined the results obtained from the two search engines to produce peptide, protein identification and semi‐quantification (spectral counting) for each sample. This process was executed as a Snakemake [30] pipeline.

The search parameters for each metaproteomic dataset, such as precursor mass tolerance and fragment mass tolerance, digesting enzymes, fixed and variable modifications, were set as described in the respective publications (Supporting Information, Section 1). When the information was not provided in the publication, the following default parameters were employed: peptides of 7–60 amino acids with a maximum of two missed cleavage sites. Peptides were grouped by both mass and sequence, and validated based on *q*‐values with a FDR of 1% at the protein level. Semi‐quantitative results were also generated using spectral counting (as calculated in PeptideShaker).

### 2.4 Post‐Processing

The results from PeptideShaker for each dataset were processed to quality filter ambiguous protein groups based on the following criteria: (i) retain only proteins which have validated PSMs; (ii) retain protein groups which were confidently identified after the removal of all human and contaminant proteins; (iii) remove ambiguous proteins which have more than one validated protein group. Finally, the post‐processed identification and semi‐quantification results were transformed into a GFF file suitable for integrating into the MGnify web interface using a custom script, which mapped the identified proteins to their corresponding assembly (see below).

### 2.5 Integration of Metaproteomics Results Within MGnify

To support visualisation of the expressed proteins in MGnify, we have enabled the browser‐upload of GFF files into the Integrative Genomics Viewer (IGV) [31] contig viewer. The GFF file contains the genomic coordinates of the expressed proteins and metaproteomics evidence such as the unique peptide‐to‐protein matches, ambiguous peptide to protein matches, and supporting evidence in the form of the number of PSMs and spectral counting semi‐quantitative information. It also provides a link to the corresponding PRIDE‐reanalysed datasets (datasets PXD032303, PXD034617 and, respectively, Table 2) where users can download the reanalysed metaproteomics results and the protein search DBs that were used to generate the results. We have developed a specific metaproteomics track view, which recognises this format of GFF allowing a tailored IGV [31] plugin to display the data.

**TABLE 2:** Summary results of the data reanalyses for the selected search DBs.

PRIDE accession	Reanalysed PRIDE dataset accession	# databases	Database size	# PSMs	# Peptides	# Proteins	# Protein groups	Unique mappings
PXD005780	PXD032303	PXD005780_DB15	240.6 MB	100,239	17,262	15,417	8169	1467
PXD003791	PXD034617	6 DBs (PXD003791_DB16)	Each < 1GB	384,631	45,506	59,613	30,444	1687
PXD020692	PXD038539	PXD020692_DB2	928.8 MB	16,951	2542	4651	1495	182

## 3 Results

**FIGURE 1:**
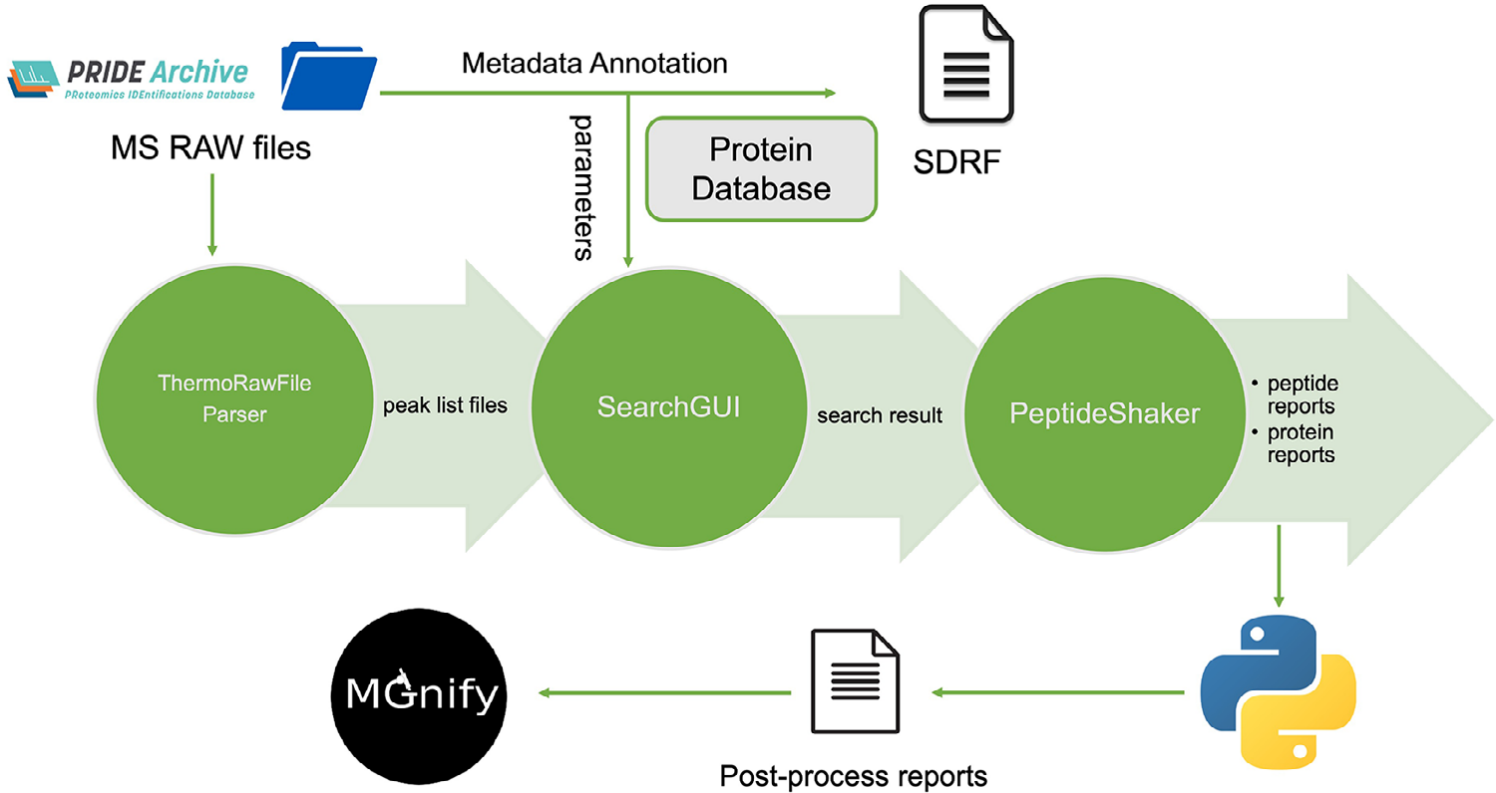
Overview of the study design and the data re‐analysis pipeline.

The selected metaproteomic datasets were benchmarked and reanalysed as described in ‘Methods’, to integrate metaproteomic, metatranscriptomic (when available) and metagenomic data. A graphical summary of the overall approach and analysis workflow is represented in Figure 1. Next, we describe the benchmarking process for the three different scenarios and selected datasets: ‘Human gut’, ‘Diabetes gut’ and ‘Marine hatchery’ datasets. The main objective was to use search DBs that, on one hand, were comprehensive enough in terms of coverage of the metaproteome, and on the other hand, were an appropriate size to make the metaproteomics analysis feasible in terms of run‐time and sensitivity. We benchmarked search DBs that had been created using different methods, which included, among others: (i) creating sample‐specific protein search DBs; (ii) combining all the predicted proteins from all the samples of the study; and (iii) integrating the above DBs with a matching subset of proteins from an appropriate biome‐specific catalogue (e.g., the UHGG catalogue for human gut data).

The final integration of results in MGnify was performed for the selected version of the search DB after performing the benchmarking.

### 3.1 Benchmarking of Search DBs for the Three Scenarios

Table 1 includes a description of the datasets used for the benchmarking study.

#### 3.1.1 Evaluating Metaproteomics Analysis of a Small Cohort of Healthy Human Gut Microbiome (‘Healthy gut’ Dataset)

First, we evaluated the smallest dataset (‘Healthy gut’), for which there is paired metagenomic and metaproteomic data. The metaproteomic dataset (accession PXD005780) consists of 15 faecal samples from a cohort of 15 healthy individuals (Table 1). From the same 15 samples, there are 37 associated metagenomic assemblies in ENA, which were uploaded by the original authors into ENA (Study accession PRJEB41181) and analysed in MGnify (Study accession MGYS00005657).

For the benchmarking process, 15 different search DBs were generated using the protein sequences from various combinations of paired metagenomic assemblies and matching genomes from the UHGG as described in Table S1. A description of the benchmarking analysis results is included in Supporting Information (Section 1.2). The resulting number of PSMs for each sample and search DB combination are available in Tables S2 and S3 along with a short description of how the DBs were created. The search DB that was chosen due to the high number of PSMs in combination with a relatively low running time was PXD005780_DB15, which contained sequences from the assembled contigs of all 15 samples grouped together. The generation of this search DB was feasible because the number of samples in the study was relatively small. Using PXD005780_DB15, an average number of 7016 PSMs were detected for each sample (Table S3).

To establish how the UHGG might perform as a substitute for a paired metagenomic dataset, we undertook reanalysis of the ‘Healthy gut’ dataset using three search DBs generated solely from UHGG‐derived sequences: PXD005780_DB12, PXD005780_DB13 and PXD005780_DB14 (see Table S1 for descriptions). The resulting three search DBs were much larger in size when compared to the other benchmarked search DBs (Table S1). The best results among these search DBs were obtained with PXD005780_DB13 (2.86 GBs in size), where an average number of 7128 PSMs were detected for each sample (a very slight increase of only 1.6% compared to the counts for PXD005780_DB15, see above). The number of identified PSMs was much lower for DB PXD005780_DB12. For the largest search DB (PXD005780_DB14, 3.69 GBs in size) the run time was prohibitively expensive.

Since the difference in number of detected PSMs between PXD005780_DB13 and PXD005780_DB15 was very small, we selected to use the paired metagenomic data approach (PXD005780_DB15), which is the current state‐of‐the‐art. All search DBs, the associated search results, as well as peptide/protein reports and processed sample reports of the reanalysis, were uploaded to PRIDE (reanalysed dataset PXD032303). Overall, 100,239 PSMs were detected in the entire dataset. Among them were 17,262 distinct peptide sequences, and a total of 8169 protein groups. In total, 1467 peptides were uniquely mapped to proteins, and 22,605 mappings were ambiguous.

### 3.2 Evaluating Metaproteomics Analyses of a Larger Dataset Involving Diabetes Type I Human Gut Microbiome

Next, we evaluated the ‘Diabetes gut’ dataset, a larger dataset comprising 36 samples and 220 assemblies (PRJEB22368), containing metagenomics and metatranscriptomics data (Table 1). Due to the larger size of the dataset, only three samples out of the total 36 were used for the benchmarking (Table S4). These samples were from the same individual (M2.4) covering three visits/time points (V1, V2, and V3). A set of 16 search DBs, utilising various combinations of paired metagenomic and metatranscriptomic assemblies and matching genomes from the UHGG, were generated for each of these three samples (full details are available in Section 1.3 of the Supporting Information). After performing the data reanalysis for these three samples, we found that the number of PSMs obtained using sample‐specific DBs (i.e., not including sequences from the UHGG) was generally higher (Table S5). DBs created from a combination of metagenomic and metatranscriptomic data performed better than those generated from either metagenomic or metatranscriptomic data alone (Table S5).

The results indicate that the preferred approach is to pool sequences from all samples in the study. However, the resulting search DB may be too large for a dataset such as this containing a larger number (36) of samples, as was the case in this instance (6.19 GB in size). One solution was to use the experimental metadata to group similar samples, and create ‘sample group’‐specific DBs (Table S6). Using this approach, we generated six search DBs (PXD003791_DB16, corresponding to six ‘sample groups’) to perform the analysis of the whole dataset (see details below) and the resulting data integration.

The six PXD003791_DB16 DBs, and all associated search results, as well as peptide/protein reports and processed sample reports of the reanalysis, were uploaded to PRIDE (reanalysed dataset PXD034617). Overall, 384,631 PSMs were detected in the entire dataset, among them were 45,506 distinct peptide sequences, and a total of 30,444 protein groups. In total, 1687 peptides were uniquely mapped to proteins.

#### 3.2.1 Evaluating Metaproteomics Analysis of a Diverse Environmental Sample (Marine Hatchery)

Next, we evaluated the ‘Marine hatchery’ dataset, comprising six samples containing paired metaproteomics and metagenomics data (Table 1, more details are available at Section 1.4 of the Supporting Information). For this study, the authors had submitted the original search DB to PRIDE, and therefore it was available for use in the benchmarking (as PXD020692_DB1). Using our experience from the previous two datasets, and as no biome‐specific genome catalogue was available at the time in MGnify, we created two types of search DB: six sample‐specific DBs (PXD020692_DB3, one per sample), and an additional search DB (PXD020692_DB2) which was generated by pooling the sequences from all six samples (Table S7). Four samples (samples 3, 11, 13 and 16) representing different experimental conditions (different pHs and sample collection days) were selected for the benchmarking.

Overall, the reanalysis produced fewer PSMs than had been seen for the human gut datasets (ratio of PSMs/spectra was 29% and 17.1% for ‘healthy gut’ and ‘diabetes gut’, respectively, and 10.5% for ‘marine hatchery’), which is expected due to the lower sequencing depth and the higher diversity of environmental samples. As the number of detected PSMs were similar across all search DBs (Table S8), we opted to make the integration approach as comparable as possible across datasets, and thus selected the search DB constructed from pooled samples (PXD020692_DB2) to carry out the analysis and data integration. PXD020692_DB2, the other search DBs and all the associated search results, peptide/protein reports and processed sample reports of the reanalysis, were uploaded to PRIDE (as the reanalysed dataset PXD038539). Overall, 16,951 PSMs were detected in the entire dataset, among them were 2542 distinct peptide sequences, and a total of 1495 protein groups. In total, 182 peptides were uniquely mapped to proteins.

For each metaproteomics analysis, the resulting peptides and proteins identified were further processed to: (i) remove the contaminant proteins (from cRAP); and (ii) include spectral counting information from the protein reports generated by PeptideShaker. A summary of this final analysis is shown in Table 2.

### 3.3 Visualisation of the Expressed Proteins on the MGnify Website

Although metaproteomics analysis identifies proteins that are actively expressed, and thus relevant to the functional characterisation of the sample, understanding the genomic context of these expressed proteins can provide greater insight into their functions, especially when they are co‐located within an operon. To enable this form of downstream analysis in the MGnify web interface, a metaproteomics track has been enabled in the ‘Contig Viewer’, part of the metagenomic assembly analysis results in MGnify. The contig viewer utilises the IGV [31] framework to visualise contigs, and the functional annotations of the proteins and non‐coding RNAs predicted in those contigs.

**FIGURE 2:**
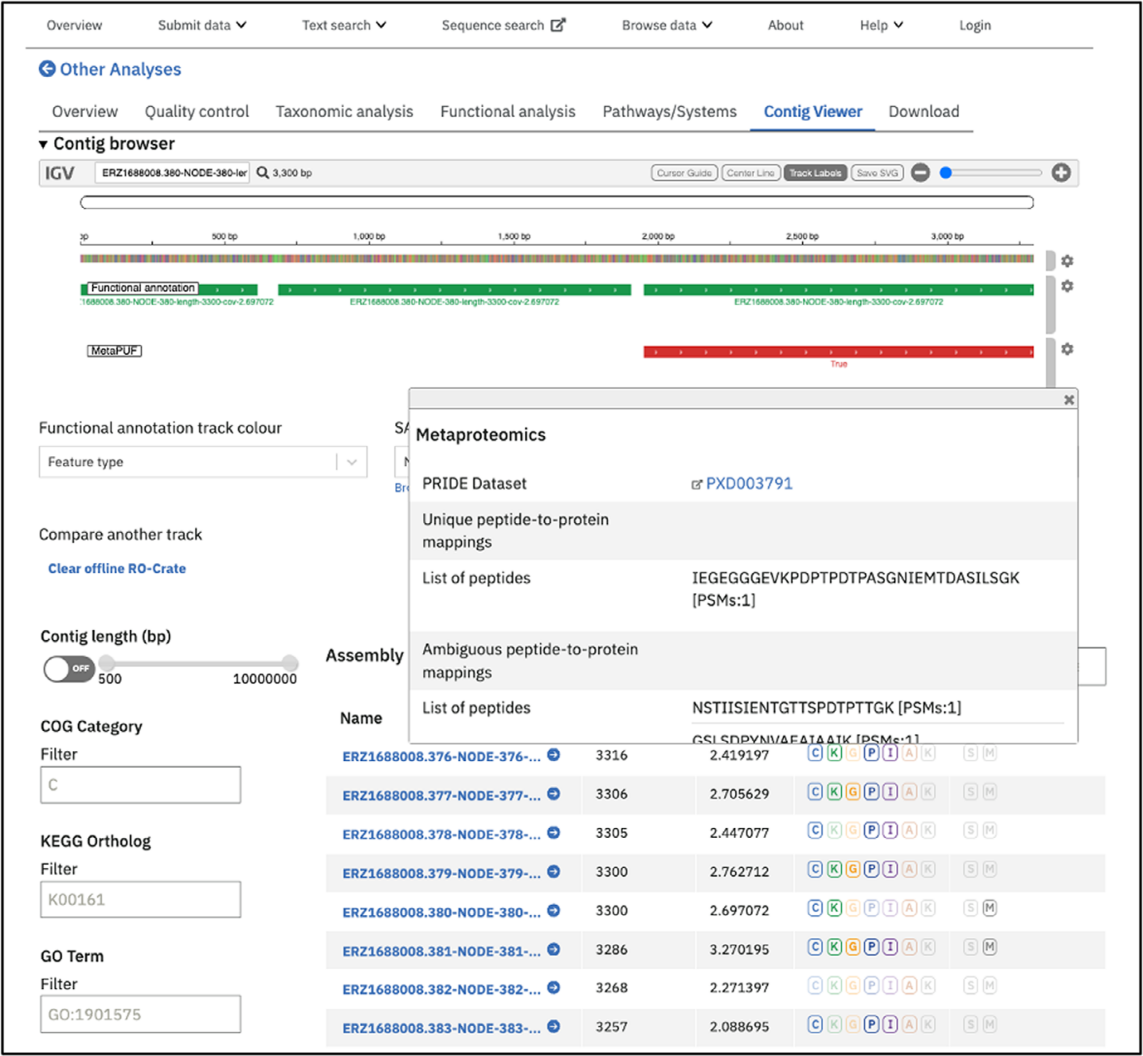
Screenshot of the MGnify contig‐viewer web interface displaying the metaproteomics track containing the expressed proteins in contig ERZ16880084.380‐Node‐380 from the MGnify analysis of study ERP104047.

To facilitate visualisation within the metaproteomics track, a script uses the results from PeptideShaker to generate a GFF formatted file containing both proteins with unambiguous peptide matches, which includes the spectral count semi‐quantitative values; and proteins with peptides that match more than one protein sequence (ambiguous peptide‐to‐protein mappings). Users can upload the GFF format file of the proteins containing unique peptide matches to highlight those proteins that were identified in the metaproteomics analysis. A mouseover event on the protein feature results in a pop‐up window providing further details on the metaproteomics analysis, including the list of unique and ambiguous peptides, the PSM spectral counts, and the start and end coordinates of the mapped proteins, an example of which is shown in Figure 2. All the datasets and results for the three studies are publicly available in PRIDE (Table 2).

### 3.4 Data Integration Open Workflow

Based on experience gained from the analysis and integration of the three datasets used in this study, we developed a generalised workflow that integrates metaproteomic and metagenomic/metatranscriptomic data derived from the same samples. The workflow supports as input either data available in PRIDE and MGnify, or the users own metagenomic assemblies and metaproteomics data, appropriately formatted according to the documentation. The workflow, lists of associated software dependencies and usage documentation are available at https://github.com/PRIDE‐reanalysis/MetaPUF. The code repository includes some example test data to help guide users in the formatting of their own data inputs, as well as a step‐through guide on running the workflow. The post‐processing GFF‐generation script is also included in the code repository to allow visualisation of data against a MGnify assembly where appropriate.

**FIGURE 3:**
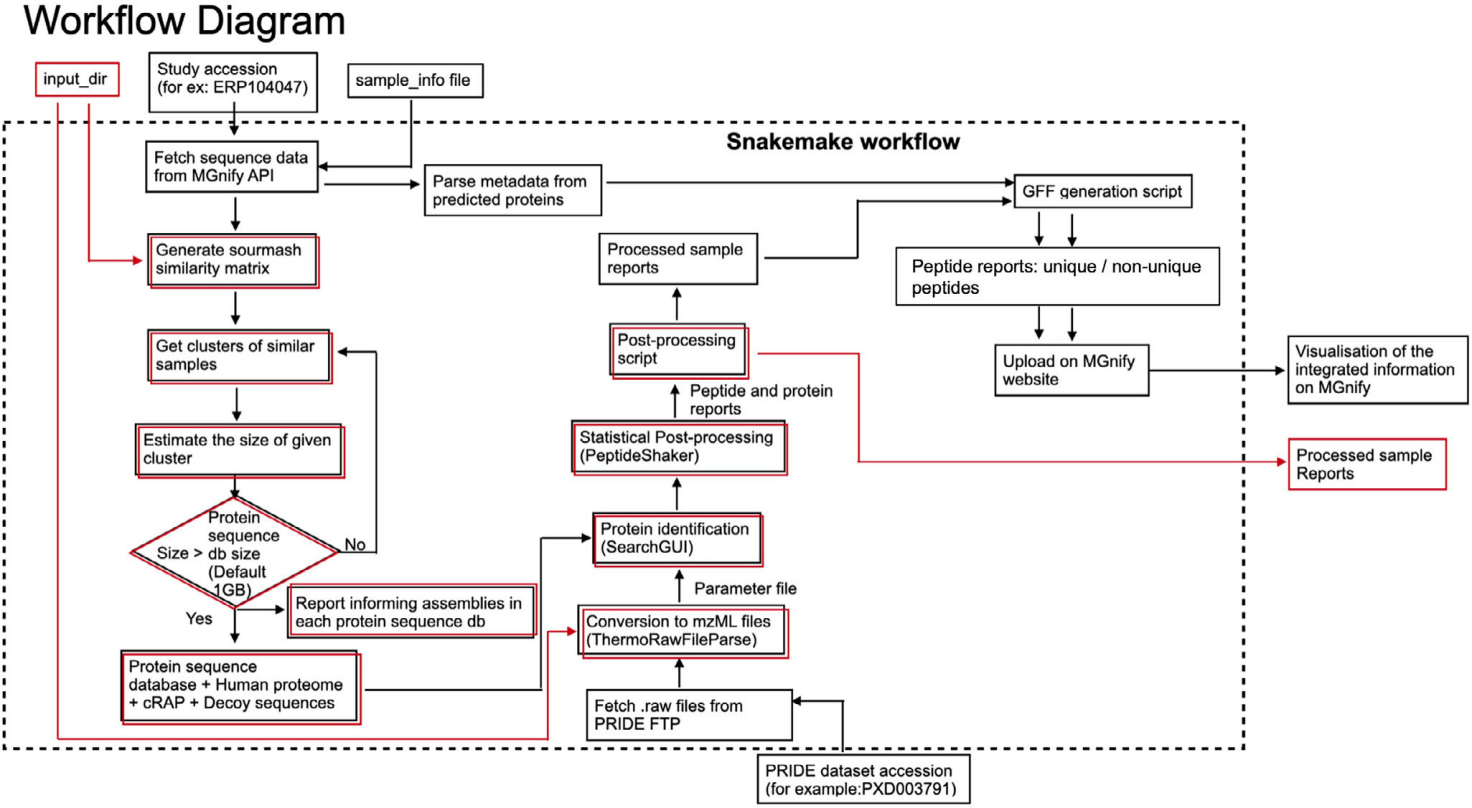
Schematic representation of the data integration workflow. The steps inside the dashed box represent the Snakemake workflow, the input and output files are indicated outside the box. The path through the workflow indicated in black represents the default scenario where a study has been assembled and analysed by MGnify, and the corresponding metaproteomics study is available in PRIDE. The workflow inputs in this scenario are the study accessions and a sample file, and the outputs are a report of the search DBs used, the results of the metaproteomics analysis and a GFF format file, which can be visualised in the MGnify web interface. The red path represents the scenario where users provide their own data (not previously analysed by MGnify and PRIDE). In this case, the input is a file path to the assembly files and proteomics raw files. The output is a report of the search DBs and the results of the metaproteomics analysis.

The workflow (schematically represented in Figure 3) consists of three sub‐workflows, involving: (i) the generation of study‐specific or sample‐specific sequence search DBs for the metaproteomics analysis. In this subworkflow sample replicates (technical and/or biological replicates) are combined prior to the generation of sequence DBs. If combining all sample‐specific sequence datasets results in a searchDB that exceeds the user‐defined maximum file size (default is 1 GB), Sourmash is used to estimate the similarity between metagenomic assemblies, thus establishing a hierarchy of related samples. The code then recursively generates search DBs using more closely related assemblies until a set of search DBs that are all below the maximum file size are defined. A report is generated detailing the groups of assemblies that correspond to the proteins found in each search DB. Each search DB is concatenated to the UniProt human reference protein (release‐2019_05), common lab contaminants (cRAP) and a dynamically generated decoy sequence DB; (ii) metaproteomics analysis using the aforementioned search DBs and the MS raw files (Thermo Fisher Scientific files) as described in the corresponding ‘Methods’ section (‘Proteomics raw data processing subsection’); and (iii) generation of GFF files required to visualise the integrated information in the MGnify web interface. In the scenario where users input their own data (which is not in MGnify), the GFF outputs of the third sub‐workflow cannot be uploaded to the MGnify web interface, but can be viewed in many common sequence viewing tools to understand the context of the expressed proteins.

## 4 Discussion

Meta‐omics data integration of different omics layers offers unparalleled opportunities to understand the structure (metagenome) and functional expression (metatranscriptome and metaproteome) of the microbiome, which plays a significant role in the health of humans and the planet. As such, there is a growing need to identify relevant datasets and provide reproducible methods to enable their integration and visualisation. Despite its unparalleled advantages, the number of publicly available paired meta‐omics dataset (metaproteomics, metagenomics and/or metatranscriptomics) is still relatively small.

The success of the metaproteomics analysis is hugely dependent on having the appropriate search DB [32]. Although we have discussed some of the issues with creating those, it is noteworthy that the diversity of the microbiota found in the sample is also going to play a huge factor in the analysis, with diverse environments such as marine requiring significantly greater sequencing depths compared to the human gut to capture the same fraction of diversity, with even more diverse environments such as soil amplifying this challenge further still.

Indeed, in this study we have used three public multi‐omics datasets with different characteristics, including the number of samples, the sampled environment (human gut and marine hatchery biomes), and the availability (or not) of paired metatranscriptomics data in addition to paired metagenomics information. A similar data integration approach for metaproteomics datasets without counterpart nucleic acid sequence information generated in the same samples would be more challenging.

In terms of data reanalysis, the logical approach would be to use well‐established collections of biome‐specific datasets as a search DB (such as in the case of human gut datasets). However, this approach can be problematic since the search DB in the case of the human gut may become very large, increasing computational analysis time and resulting in a lower sensitivity due to the FDR statistical thresholds (as demonstrated in the ‘Healthy gut’ dataset). As these metagenomics datasets become more comprehensive, with rich sample metadata, one approach could be the creation of more specific subsets that better reflect the characteristics of the metaproteomic samples, such as geographical range or disease state. An alternative approach would be to produce search DBs that are subsampled based on sequence redundancy or taxonomy to provide a final search DB, within a specific size‐range. The DB size limitation was implemented in the ‘Diabetes Gut’ dataset and in the downloadable workflow.

Furthermore, it is important to note that while the metagenomics/metatranscriptomics generated search DB could be made more comprehensive with our approaches, increasing the DB size does not have a positive impact on the results if the DB is too big compared to the metaproteomics dataset. As novel mass spectrometer machines and techniques are being developed and implemented for metaproteomics [33], it will lead to deeper analyses and provide better peptides/proteins coverage. We expect this improvement to better match our improved DBs and ultimately lead to much higher identification rates. Additionally, more tailored (meta)proteomics analysis approaches could also be explored, such as the use of two‐step search DB methodology [34] or rescoring using MS/MS spectra fragmentation predictions as the basis (e.g., [35]), among other options. Finally, a future objective would be to enable the tracing back of each peptide's experimental evidence from the MGnify web interface to the original MS spectra in PRIDE using Universal Spectrum Identifiers [36].

As multi meta‐omics datasets will become increasingly available, there is a dire need for frameworks and established data resources where users can link their different types of data, analyse it in an integrated manner, and explore and visualise it all together. In line with other studies, we have demonstrated that the use of paired samples usually offers the best results for the analysis and integration of metaproteomics data. With this in mind, undoubtedly the major hurdle to overcome for multi‐omics data is the more systematic linkage between the different data types in public data resources. Current solutions are restricted to connecting datasets via publications (e.g., using the PubMed ID), or using more automated accession matches as offered by EuropePMC [37]. In the near future, the use of common sample identifiers will be essential. Submission of nucleic acid sequences to ENA (or to any member of the International Nucleotide Sequence Database Consortium, INSDC) requires that the samples are registered with the BioSamples DB [38]. This already offers a straightforward way to connect metagenomics and metatranscriptomics datasets derived from the same sample. The propagation of BioSamples identifiers to metaproteomics would rely on both the data generator to ensure that samples are assigned a BioSample accession at an early stage, prior to going to the respective omics sequencing facility, and developments in PRIDE to require BioSamples accessions as a mandatory field in deposition criteria (at the moment this information is optional in dataset submissions to PRIDE).

The availability of linked meta‐omics datasets from different omics layers has great potential for different applications. One promising one is to help characterise PUFs (Proteins of Unknown Function), which account for a substantial portion of metaproteomics data. This is important from the point of view of knowing which ones should be put on a priority list for functional validation. Indeed, focusing on conserved expressed PUFs across different samples and conditions offers a strategic starting point, as these proteins may play critical biological roles in the microbiome. By analysing, for example, their primary sequence, occurrence and abundance, researchers can begin to unlock the functional significance of these proteins.

Here, we introduce an approach for the systematic integration of public meta‐omics datasets generated in multi‐omics studies, involving metaproteomics and the corresponding metagenomic and/or metatranscriptomic data, to produce specific search DBs for the analysis of the metaproteomics data. The workflow is implemented in MGnify and PRIDE, two world‐leading and established resources in the field, allowing streamlined linking between the different omics datasets and repositories. We provide a method for the representation of metaproteomics data in the MGnify web interface, to improve clarity for non‐experts in proteomics. Currently, we have broadly categorised peptide matches into those that match proteins uniquely (unambiguous), and those that match multiple proteins (ambiguous). This may reflect an underestimate of proteins that are expressed, and we will review this approach as feedback is received from users.

## 5 Conclusions

We believe that this study represents the first step toward systematic data integration of multi‐meta‐omics datasets between two key biological data resources. As the number of microbiome multi‐omics datasets increases, there will be a growing need for such integration, as well as easier access to data and visualisations.

## Conflicts of Interest

The authors have declared no conflict of interest.

## Supporting information



Supporting information

## Data Availability

This study reused three public datasets in the PRIDE database (PXD005780, PXD003791 and PXD020692). The reanalysed versions of the three datasets have also been made available via PRIDE (datasets PXD032303, PXD034617 and PXD038539).
