# Supplementary material for: An Approach to Integrate Metagenomics, Metatranscriptomics and Metaproteomics Data in Public Data Resources
Source: Proteomics. 2025 Apr 28;25(17-18):33–42. doi: 10.1002/pmic.202500002 (PMC12456259; doi:10.1002/pmic.202500002)
Supplement: Supplementary file 1 — Supporting information [file PMIC-25--s001.docx]

**Supplementary Information: “An approach to integrate metagenomics, metatranscriptomics and metaproteomics data in public data resources“**

**1) Benchmarking of metaproteomics datasets using different search databases (search DBs)**

**1.1) Search parameters for the metaproteomics data analysis**

Three datasets were analysed on a Linux high-performance computing cluster with 150 GB of memory and 4 CPUs. Input parameters for each dataset when searching, such as MS1 and MS2 tolerances, digesting enzymes, fixed and variable modifications, were set as described in their respective publications.

The common parameters in these three datasets were: (i) Fixed modifications were all set as Cysteine carbamidomethylation, and variable modification was oxidation of methionine (M); (ii) The FDR (False Discovery Rate) at the PSM (Peptide Spectrum Match) and protein levels were set to 1%; and (iii) Peptides were allowed to have a maximum of two missed cleavages.

The differences among these three datasets were: for PXD005780 (‘Healthy gut’), peptide length was set from 5-50 amino acids, and the precursor mass tolerance was set to the value of 10 ppm and fragment mass tolerances were set to 0.02 Da; for PXD003791 (‘Diabetes gut’), there was an additional variable modification, which was Acetylation of the protein N-terminus, peptide length was set from 7-60 amino acids, and the precursor mass tolerance was set to the value of 10 ppm and fragment mass tolerances were set to 0.02 Da; and for PXD020692 (‘Marine hatchery’), peptide length was set from 6-60 amino acids, and the precursor mass tolerance was set to the value of 20 ppm and fragment mass tolerances were set to 0.02 Da.

**1.2) Analysis of the ‘Healthy gut’ dataset (PXD005780)**

Ultimately, 15 search databases were constructed (PXD005780_DB1 to PXD005780_DB15) and benchmarked in the analysis of PXD005780. Briefly, paired metagenomic datasets were assembled and protein sequences predicted as described in the ‘Methods’ section of main text. Sourmash was used to compare assembled contigs with the Unified Human Gastrointestinal Genome (UHGG) catalogue to identify “matching genomes” for use in supplementing search DBs. Descriptions of how each search DB was constructed are provided in Supplementary Table 1.

| **Database Name** | **DB size (MB)** | **Description** |
| --- | --- | --- |
| PXD005780_DB1 | 84 | Contains sequences from all matching genomes from the UHGG, clustered at 100% sequence identity. |
| PXD005780_DB2 | 87 | Contains sequences from the paired metaG assemblies, clustered at 100% sequence identity. |
| PXD005780_DB3 | 85 | Contains sequences from all matching representative genomes from the UHGG, clustered at 100% sequence identity. |
| PXD005780_DB4 | 159 | Contains sequences from all matching representative genomes from the UHGG. |
| PXD005780_DB5 | 155 | Contains sequences from all genomes from the UHGG. |
| PXD005780_DB6 | 159 | Contains sequences from the paired metaG assemblies. |
| PXD005780_DB7 | 82 | Contains sequences from all matching genomes from the UHGG, clustered at 98% sequence identity. |
| PXD005780_DB8 | 80 | Contains sequences from all matching genomes from the UHGG, clustered at 97% sequence identity. |
| PXD005780_DB9 | 79 | Contains sequences from all matching genomes from the UHGG, clustered at 96% sequence identity. |
| PXD005780_DB10 | 78 | Contains sequences from all matching genomes from the UHGG, clustered at 95% sequence identity. |
| PXD005780_DB11 | 72 | Contains sequences from all matching genomes from the UHGG, clustered at 90% sequence identity. |
| PXD005780_DB12 | 484 | Contains sequences from all representative genomes from the UHGG defined as originating from European populations. |
| PXD005780_DB13 | 2,860 | Contains sequences from all pan-genomes from the UHGG defined as originating from European populations. |
| PXD005780_DB14 | 3,686 | Contains sequences from all genomes from the UHGG defined as originating from European populations. |
| PXD005780_DB15 | 152 | same as DB6 with a difference that all the runs from samples were pooled and then processed similarly as DB6. |

**Supplementary Table 1.** Description of the 15 benchmarking databases used for the analysis of the ‘Healthy gut’ dataset (PXD005780).

In the first round, 11 different search DBs (PXD005780_DB1 to PXD005780_DB11), concatenated with the cRAP contaminants database, were constructed and used to analyse each of the 15 samples. Running times were similar for DB1 to DB3 and DB7 to DB11, with each sample taking around 35 min to run, whereas DB4, DB5 and DB6 took around 20 minutes longer. As DB6 provided the highest number of PSM, an average of 3,957 PSMs, it was selected for use in further analysis.

| **Database name** | **S1** | **S2** | **S3** | **S4** | **S5** | **S6** | **S7** | **S8** | **S9** | **S10** | **S11** | **S12** | **S13** | **S14** | **S15** | ***Mean*** | ***Running time*** |
| --- | --- | --- | --- | --- | --- | --- | --- | --- | --- | --- | --- | --- | --- | --- | --- | --- | --- |
| **PXD005780_DB1** | 5967 | 2031 | 4305 | 4546 | 4362 | 3050 | 3915 | 3156 | 3351 | 4233 | 1989 | 4046 | 5657 | 3533 | 2823 | 3798 | 37 min |
| **PXD005780_DB2** | 6030 | 2088 | 4291 | 4780 | 4472 | 3091 | 3882 | 3163 | 3452 | 4249 | 2271 | 4452 | 5737 | 3461 | 2829 | 3883 | 39 min |
| **PXD005780_DB3** | 5898 | 2100 | 4303 | 4647 | 4343 | 3040 | 3956 | 3161 | 3358 | 4209 | 2185 | 4465 | 5744 | 3462 | 2839 | 3843 | 39 min |
| **PXD005780_DB4** | 6017 | 2202 | 4558 | 4780 | 4438 | 3153 | 3932 | 3189 | 3317 | 4399 | 2140 | 4451 | 5743 | 3580 | 2897 | 3920 | 57 min |
| **PXD005780_DB5** | 6095 | 2188 | 4445 | 4669 | 4430 | 3163 | 3900 | 3179 | 3457 | 4426 | 2010 | 4268 | 5816 | 3445 | 2939 | 3895 | 53 min |
| **PXD005780_DB6** | 6094 | 2152 | 4488 | 4828 | 4521 | 3146 | 3951 | 3206 | 3369 | 4408 | 2187 | 4515 | 5927 | 3621 | 2940 | 3957 | 65 min |
| **PXD005780_DB7** | 5942 | 2014 | 4257 | 4541 | 4355 | 3021 | 3831 | 3111 | 3303 | 4178 | 2007 | 4093 | 5648 | 3433 | 2835 | 3771 | 39 min |
| **PXD005780_DB8** | 5870 | 2028 | 4177 | 4539 | 4377 | 3022 | 3950 | 3112 | 3313 | 4138 | 2001 | 4064 | 5559 | 3406 | 2773 | 3755 | 36 min |
| **PXD005780_DB9** | 5776 | 2012 | 4166 | 4432 | 4284 | 2989 | 3914 | 3107 | 3174 | 4153 | 1926 | 3871 | 5528 | 3412 | 2702 | 3696 | 34 min |
| **PXD005780_DB10** | 5759 | 1966 | 4016 | 4424 | 4200 | 2937 | 3836 | 3049 | 3211 | 3951 | 1861 | 3806 | 5462 | 3423 | 2642 | 3636 | 32 min |
| **PXD005780_DB11** | 5394 | 1797 | 3847 | 4033 | 3912 | 2689 | 3531 | 2928 | 2945 | 3675 | 1688 | 3473 | 4935 | 3129 | 2393 | 3358 | 31min |

**Supplementary Table 2.** Number of ​​PSMs identified for each sample and search DB combination in the first round of benchmarking of PXD005780.

In the second round we added the UniProt human reference proteome (release-2019_05, including isoforms, 95,915 sequences) to the search DBs, to account for host contamination within the microbiome samples. Five search DBs were compared in the second round: PXD005780_DB6 (the best performing search DB from the first round), PXD005780_DB15 (constructed in a similar was to PXD005780_DB6 but pooling all runs from samples) and an additional three search DBs utilising only the data from the MGnify Genomes human gut catalogue (UHGG) (PXD005780_DB12, PXD005780_DB13, and PXD005780_DB14). This approach was included to evaluate the use of genome catalogues in cases where no matched metagenomic data existed.

PXD005780_DB6 gave an average search result of 6,675 PSMs, whereas PXD005780_DB15 gave an average of 7,016 PSMs. In the case of UHGG-based search DBs, the best results were obtained with PXD005780_DB13 (2.6 GBs in size), where an average number of 7,128 PSMs were detected. However the running time increased to an average of 361 mins. When the largest search DB from UHGG (PXD005780_DB14) was used, running times increased by a factor more than 10, resulting in the searches not completing within the permitted time frame. Therefore, results are only provided for the first 4 samples that were run.

| **Database name** | **S1** | **S2** | **S3** | **S4** | **S5** | **S6** | **S7** | **S8** | **S9** | **S10** | **S11** | **S12** | **S13** | **S14** | **S15** | **Mean** | **Average Running time** |
| --- | --- | --- | --- | --- | --- | --- | --- | --- | --- | --- | --- | --- | --- | --- | --- | --- | --- |
| **PXD005780_DB6** | 8,203 | 8061 | 8596 | 7265 | 6124 | 5535 | 5913 | 5646 | 7248 | 7357 | 5894 | 6613 | 8046 | 5392 | 4238 | 6675 | 83 min |
| **PXD005780_DB12** | 5352 | 6877 | 5843 | 5199 | 4743 | 4374 | 4053 | 4313 | 5698 | 5259 | 4549 | 4359 | 6248 | 4128 | 3038 | 4936 | 251 min |
| **PXD005780_DB13** | 7402 | 7424 | 8136 | 8124 | 7423 | 6515 | 6642 | 6704 | 7535 | 7520 | 5446 | 7417 | 8286 | 7037 | 5308 | 7128 | 361 min |
| **PXD005780_DB14** | 8145 | 7659 | 8526 | 8633 | NA | NA | NA | NA | NA | NA | NA | NA | NA | NA | NA | NA | NA |
| **PXD005780_DB15** | 8728 | 8026 | 8905 | 7660 | 6686 | 5728 | 6477 | 5823 | 7607 | 7431 | 5972 | 6927 | 8427 | 6102 | 4738 | 7016 | 98 min |

**Supplementary Table 3.** Number of ​​PSMs identified for each sample and search DB combination in the second round of benchmarking of PXD005780.

Note: Only four counts are available for DB14 as the pipeline did not complete due to the large size of this database (~3.7 GBs).

**1.3) Analysis of the ‘Diabetes gut’ dataset (PXD003791).**

Overall, 16 search DBs were created and benchmarked. Some of the different approaches used to construct the databases were: (i) perform a co-assembly of metagenomics and metatranscriptomics samples; (ii) concatenate the predicted proteins from metagenomics and metatranscriptomics samples; (iii) use the sourmash comparison module on all the assemblies, which clustered the metagenomics and metatranscriptomics assemblies separately; and (iv) generate ‘sample group’ specific search DBs, where the first group (PXD003791_DB1 to PXD003791_DB12) were generated using the Prodigal for protein prediction, and the second group (PXD003791_DB13 to PXD003791_DB16) were generated using a combined gene caller (Prodigal and FragGeneScan) (see ‘Methods’). A detailed description of each database can be found in Supplementary Table 4.

Only three samples out of the total 36 were used for the benchmarking in this case. These samples were from the same individual (M2.4) covering three visits/time points (V1, V2 and V3). Sample-specific search DBs were created for these three samples. In all cases, cRAP (contaminants) and the UniProt human reference proteome (release-2019_05, including isoforms, 95,915 sequences) were appended to those search DBs. In addition, some of the search DBs included the protein sequences coming from the UHGG (see Supplementary table 4 for details).

| **Database Name** | **Database code** | **DB size (MB)** | **Description** |
| --- | --- | --- | --- |
| PXD003791_DB1 | UHGG_MG | 563 | Contains sequences from the metaG assemblies specific to the sample, along with a matching subset of proteins from the UHGG. |
| PXD003791_DB2 | UHGG_MT | 175 | Contains sequences from the metaT assemblies specific to the sample, along with a matching subset of proteins from the UHGG |
| PXD003791_DB3 | UHGG_cluster_MG | 730 | Contains sequences from the metaG assemblies specific to all samples from an individual (in this case 3 samples), along with a matching subset of proteins from the UHGG |
| PXD003791_DB4 | UHGG_cluster_MT | 210 | Contains sequences from the metaT assemblies specific to all samples from an individual (in this case 3 samples), along with a matching subset of proteins from the UHGG |
| PXD003791_DB5 | MG_MT_Coassemble | 186 | Contains sequences from co-assembly of the metaG and metaT specific to the sample |
| PXD003791_DB6 | MG_MT_Concat | 570 | Contains sequences from metaG assembly and metaT assembly, specific to the sample |
| PXD003791_DB7 | NonUHGG_MG | 96 | Contains sequences from the metaG assemblies specific to the sample |
| PXD003791_DB8 | NonUHGG_MT | 52 | Contains sequences from the metaT assemblies specific to the sample |
| PXD003791_DB9 | NonUHGG_cluster_MG | 143 | Contains sequences from the metaG assemblies specific to all samples from an individual (in this case 3 samples) |
| PXD003791_DB10 | NonUHGG_cluster_MT | 57 | Contains sequences from the metaT assemblies specific to all samples from an individual (in this case 3 samples) |
| PXD003791_DB11 | NonUHGG_MG_MT_​​Coassemble | 103 | the DB that contains MG and MT co-assemblies from the sample itself, per sample per database |
| PXD003791_DB12 | Sample_specific | 124 | the DBs that contain MG and MT assemblies together from the sample itself, but generated by the MGnify pipeline, per sample per database |
| PXD003791_DB13 | MGnify_Concate | 1,946 | One DB that contains all assemblies from the 36 samples (MG and MT, which were combined firstly), clustered at 97% similarity of sourmash hashes; |
| PXD003791_DB14 | MGnify_cluster_MG | 784 | One database, containing all MG assemblies from these 36 samples clustered at 60% similarity of sourmash hashes. In order to reduce the size of the generated DB to less than 1 GB (for making the proteomics analysis possible), mmseqs clustered @99%, resulting to a 784 MG database; |
| PXD003791_DB15 | MGnify_cluster_MT | 286 | One database, containing all MT assemblies (uploaded in year 2021, 11 MT assemblies in total) clustered at 60% similarity of sourmash hashes; |
| PXD003791_DB16 | MGnify_group | ~340 | 6 databases, sample wise clustering of all the MG and MT assemblies from the corresponding samples shown in supplementary table 6 @60% similarity of sourmash hashes. |

**Supplementary Table 4.** Description of the initial 16 different search DBs used for the benchmarking of the ‘diabetes gut’ dataset (PXD003791). The database codes can be used to identify corresponding pairs of search DBs where one does include matching sequences from the UHGG and one does not (as indicated by the NonUHGG prefix in the database code).

After performing the data reanalysis, we found that the best option was to combine both sample-specific metagenomic and metatranscriptomic data at the assembly level, if both were available. Finally, databases created using metatranscriptomic data only provided increased numbers of PSMs compared to those using metagenomics data only.

| **Samples** | **DB (including human and cRAP)** | **DB Size (MB)** | **#PSMs** | **#Protein Groups** | **Running time** |
| --- | --- | --- | --- | --- | --- |
| M2.4-V1 | PXD003791_DB1 | 563 | 7,961 | 2,153 | 12.1 h |
|  | PXD003791_DB2 | 175 | 8,243 | 2,192 | 4.4 h |
|  | PXD003791_DB3 | 730 | 7,952 | 2,113 | 14.1 h |
|  | PXD003791_DB4 | 210 | 8,610 | 2,135 | 5.2 h |
|  | PXD003791_DB5 | 588 | 8,375 | 2,236 | 12.2 h |
|  | PXD003791_DB6 | 570 | 8,192 | 2,252 | 11.3 h |
|  | PXD003791_DB7 | 96 | 8,401 | 2,027 | 3.1 h |
|  | PXD003791_DB8 | 52 | 9,369 | 2,215 | 2.3 h |
|  | PXD003791_DB9 | 143 | 8,395 | 2,163 | 4.1 h |
|  | PXD003791_DB10 | 57 | 8,685 | 2,154 | 2.4 h |
|  | PXD003791_DB11 | 103 | 8,740 | 2,237 | 3.2 h |
|  | PXD003791_DB12 | 124 | 9,255 | 2,167 | 3.3 h |
|  | PXD003791_DB13 | 1,946 | 5,576 | 1,702 | 13.3 h |
|  | PXD003791_DB14 | 784 | 7,944 | 2,201 | 6.6 h |
|  | PXD003791_DB15 | 286 | 9,349 | 2,586 | 5.4 h |
|  | PXD003791_DB16 | 505 | 9,055 | 2,610 | 6.1 h |
| M2.4-V2 | PXD003791_DB1 | 529 | 7,383 | 2,630 | 10.6 h |
|  | PXD003791_DB2 | 189 | 7,583 | 2,567 | 4.5 h |
|  | PXD003791_DB3 | 730 | 7,523 | 2,722 | 14.3 h |
|  | PXD003791_DB4 | 210 | 7,249 | 2,415 | 5.0 h |
|  | PXD003791_DB5 | 546 | 7,898 | 2,779 | 11.0 h |
|  | PXD003791_DB6 | 538 | 7,714 | 2,814 | 11.0 h |
|  | PXD003791_DB7 | 87 | 7,806 | 2,565 | 2.3 h |
|  | PXD003791_DB8 | 56 | 8,833 | 2,757 | 2.3 h |
|  | PXD003791_DB9 | 143 | 8,183 | 2,731 | 3.6 h |
|  | PXD003791_DB10 | 57 | 8,086 | 2,577 | 2.3 h |
|  | PXD003791_DB11 | 95 | 8,675 | 2,930 | 3.1 h |
|  | PXD003791_DB12 | 124 | 8,837 | 2,717 | 3.3 h |
|  | PXD003791_DB13 | 1,946 | 5,444 | 2,217 | 13.3 h |
|  | PXD003791_DB14 | 784 | 7,883 | 2,916 | 6.6 h |
|  | PXD003791_DB15 | 286 | 8,729 | 3,122 | 5.4 h |
|  | PXD003791_DB16 | 505 | 8,485 | 3,040 | 6.1 h |
| M2.4-V3 | PXD003791_DB1 | 534 | 7,131 | 2,090 | 11.2 h |
|  | PXD003791_DB2 | 84 | 7,398 | 1,916 | 4.1 h |
|  | PXD003791_DB3 | 730 | 7,100 | 2,094 | 15.5 h |
|  | PXD003791_DB4 | NA | NA | NA | NA |
|  | PXD003791_DB5 | NA | NA | NA | NA |
|  | PXD003791_DB6 | 537 | 7,177 | 2,069 | 16.2 h |
|  | PXD003791_DB7 | 96 | 6,867 | 1,840 | 3.3 h |
|  | PXD003791_DB8 | 51 | 7,236 | 1,801 | 2.5 h |
|  | PXD003791_DB9 | 143 | 7,423 | 2,023 | 4.3 h |
|  | PXD003791_DB10 | NA | NA | NA | NA |
|  | PXD003791_DB11 | 104 | 7,537 | 2,106 | 3.4 h |
|  | PXD003791_DB12 | 124 | 8,467 | 2,095 | 3.3 h |
|  | PXD003791_DB13 | 1,946 | 6,164 | 2,230 | 13.3 h |
|  | PXD003791_DB14 | 784 | 7,646 | NA | 6.6 h |
|  | PXD003791_DB15 | 286 | 8,734 | 2,354 | 5.4 h |
|  | PXD003791_DB16 | 505 | 8,665 | NA | 6.1 h |

**Supplementary Table 5.** Summary of the analysis results for different search DB for the ‘Diabetes gut’ dataset (PXD003791).

As learned from the benchmarking of dataset PXD005780, the preferred approach is to pool all sequences from all samples included in a given study. However, when working with a dataset such as this, containing a larger number of samples (36), the resulting search DB from pooling all metagenomic and metatranscriptomic samples would be too large (6.19 GBs). A solution is to use the experimental metadata, to group similar samples, and create “sample group” specific databases.

It is also possible to carry out such a grouping without using sample metadata, for example where sample metadata does not exist. For this we used the sourmash sub workflow to iteratively compare and combine samples, grouping them into sets based on similarity, where the resulting search DBs were <1GB in size. This resulted in six search DBs (Supplementary Table 6). It is worth noting that the resulting search DBs contained groups of samples from the same individuals and families, as would be expected. These six search DBs (PXD003791_DB16, corresponding to six “sample groups”) were used to perform the analysis of the whole dataset.

| **Sample Groups** | **DB size (MB)** | **Samples** | **Sample names** |
| --- | --- | --- | --- |
| **Group 1** | 296.3 | S1,S2,S3,S10,S11 (5 samples) | M1.1-V1,M1.1-V2,M1.1-V3,M1.4-V2,M1.4-V3 |
| **Group 2** | 207.3 | S4,S5,S6 (3 samples) | M1.2-V1,M1.2-V2,M1.2-V3 |
| **Group 3** | 176.7 | S7,S8,S9 (3 samples) | M1.3-V1,M1.3-V2,M1.3-V3 |
| **Group 4** | 419.3 | S15,S16,S23,S24,S25,S26,S27 (7 samples) | M2.2-V2,M2.2-V3,M2.5-V1,M2.5-V2,M3.3-V3,M3.4-V2,M3.5-V1 |
| **Group 5** | 488.9 | S12,S13,S14,S17,S18,S19,S20,S21,S22 (9 samples) | M2.1-V2,M2.1-V3,M2.2-V2,M2.3-V1,M2.3-V2,M2.3-V3,**M2.4-V1,M2.4-V2,M2.4-V3** |
| **Group 6** | 446.9 | S28,S29,S30,S31,S32,S33,S34,S35,S36 (9 samples) | M4.1-V1,M4.1-V2,M4.1-V3,M4.3-V1,M4.4-V1,M4.4-V3,M4.5-V3,M4.6-V2,M4.6-V3 |

**Supplementary Table 6.** Relationships between the samples, sample groups and corresponding search DB sizes. The Group 5 search DB was used in the benchmarking as it contained the data for the three samples being used, specifically M2.4-V1, M2.4-V2 and M2.4-V3 which are highlighted in bold.

**1.4) Analysis of the ‘Marine hatchery’ dataset (PXD020692)**

Four samples (out of the total six) and three search DB were used for the benchmarking. In this case, the original search DB used by the authors was also submitted to PRIDE (PXD020692_DB1). Additional search DBs were constructed using sample specific databases (PXD020692_DB3). Additionally PXD020692_DB2 was constructed from pooling the six samples together.

| **Database Name** | **Database code** | **DB size** | **Description** |
| --- | --- | --- | --- |
| PXD020692_DB1 | Proteins_MGM | 510 MB | Search database submitted by the original authors to PRIDE |
| PXD020692_DB2 | Proteins_Pooled | 886 MB | Unique database containing the pooled 6 metagenomics assemblies, as generated by the MGnify pipeline |
| PXD020692_DB3 | Proteins_Sample | Avg. | Sample specific databases: each sample has a corresponding search DB |

**Supplementary Table 7.** Description of the search DBs used for the benchmarking of the ‘marine hatchery’ dataset (PXD020692).

| **Database Name** | **Samples (Number of PSMs)** | | | | |
| --- | --- | --- | --- | --- | --- |
|  | **S3** | **S11** | **S13** | **S16** | **Average** |
| PXD020692_DB1 | 1,803 | 1,615 | 1,411 | 1,201 | 1,507.5 |
| PXD020692_DB2 | 1,797 | 1,644 | 1,413 | 1,105 | 1,489.8 |
| PXD020692_DB3 | 1,468 | 1,662 | 1,445 | 1,224 | 1,449.8 |

**Supplementary Table 8.** Number of ​​PSMs identified in the analysis of the ‘Marine hatchery’ dataset (PXD020692). Four samples (S3, S11, S13 and S16) were used for the benchmarking.

The numbers of detected PSMs were similar per benchmarked search DB. However, similarly to what we did in the other cases, we opted to use the search DB constructed from pooling all samples (PXD020692_DB2), for the data integration between PRIDE and MGnify.
